# Successful treatment of metastatic pheochromocytoma in the spine with cement augmentation

**DOI:** 10.1097/MD.0000000000005892

**Published:** 2017-01-27

**Authors:** Siyi Cai, Xiangyi Kong, Chengrui Yan, Yong Liu, Xi Zhou, Guixing Qiu

**Affiliations:** a Department of Orthopaedic Surgery; b Department of Neurosurgery, Peking Union Medical College Hospital, Peking Union Medical College and Chinese Academy of Medical Sciences, Beijing, China; c Department of Anesthesia, Critical Care and Pain Medicine, Massachusetts General Hospital, Harvard Medical School, Harvard University, Boston, MA.

**Keywords:** cement, metastatic pheochromocytoma, osteoplasty, spine

## Abstract

Metastatic pheochromocytoma in the spine is rare, and there is no standard curative management. Treatment via open surgery is often risky in the perioperative period, while osteoplasty by cement augmentation is a less invasive option.

We describe 2 patients with recurrence of pheochromocytoma involving the spine and the pelvis who were successfully treated with osteoplasty by cement augmentation. A 31-year-old female underwent cement augmentation for a pelvic lesion 6 months after the resection and screw fixation of an L3 lesion. A 58-year-old male underwent cement augmentation to directly destroy the functional tumor, with a surgical decompression 6 months later. Both patients showed appropriate destruction of the tumor, adequate pain relief, and the decreased release of catecholamine from metastatic lesions.

Osteoplasty by cement augmentation may be a treatment option for patients with metastatic pheochromocytoma who cannot undergo appropriate surgery or decline surgery. This represents a safe approach to sustainably relieve pain and stabilize vertebral bodies with metastatic malignant pheochromocytoma.

## 1 Introduction

Pheochromocytomas are rare tumors with an incidence of 1 per 100,000 per year, 10% to 20% of which will develop to malignant extraadrenal pheochromocytoma.^[1]^ Malignancy is defined by the presence of metastases and local invasion rather than by histological criteria. Spinal metastases are extremely rare, with various clinical presentations based on the primary lytic bone lesions that cause destruction or compression of the spinal cord, clinically manifesting as pain or neurological deficit.^[1,2]^ Previous reports investigating treatment methods for malignant tumor in the spine have studied preoperative embolization of vascular supply, chemotherapy and/or radiotherapy, and/or tumor resection,^[3]^ and concluded that open curative resection of the sectional lesion was effective. However, open surgery carries marked challenges including hemodynamic drastic fluctuation induced by catecholamine secretion from the tumor, blood loss caused by lesion destruction, and spinal stabilization.^[4]^ Herein, we present 2 cases in which pheochromocytoma of the spine was successfully treated via osteoplasty by cement augmentation for the first time; this method deactivated the neuroendocrine systems, reduced perioperative blood loss, and stabilized the spine. Written informed consent was obtained from the patient for publication of this article. A copy of the written consent is available for review by the editors of MEDICINE. Because this article does not involve any human or animal trials, there is no need to conduct special ethic review and the ethical approval is not necessary.

## 2 Case reports

### 2.1 Case 1

#### 2.1.1 History and examination

A 31-year-old female with a 17-year history of hypertension (maximum blood pressure (BP) ≤200/120 mm Hg) underwent surgical resection of adrenal pheochromocytoma 5 years previously. Postoperative BP returned to normal with no evidence of distant metastasis during a 4-year follow-up. However, the patient experienced severe paroxysmal headaches provoked by hypertensive peaks, moderate low back pain and numbness of the right lower limb for 1 year before being referred to our hospital.

**Figure 1:**
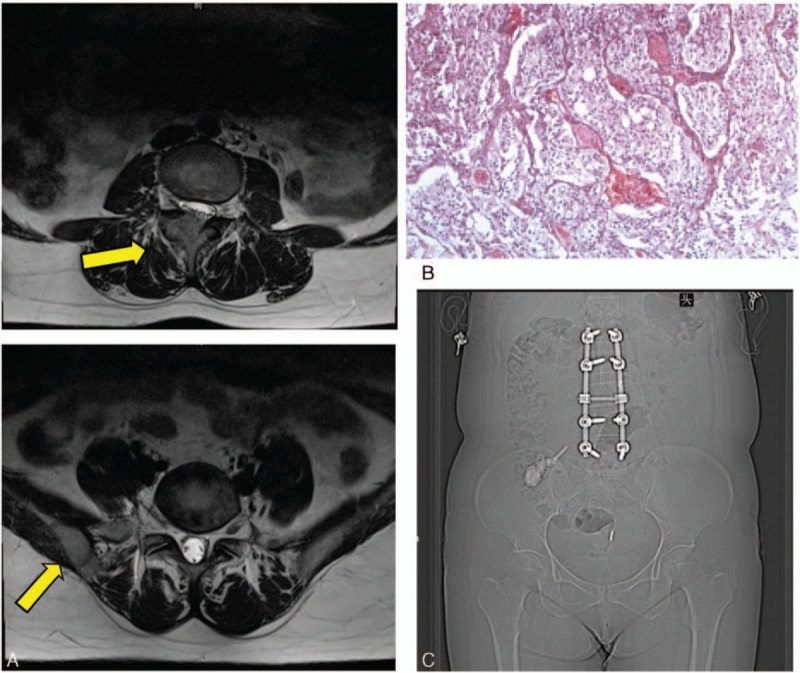
Case 1. (A) MRI showing enhanced lesions in the right pedicle and facet of L3 and the right ilium. (B) Hematoxylin and eosin-stained histological sections from the biopsy specimen confirmed extraadrenal pheochromocytoma. (C) The location of 2-step surgery. In the first operation, paraganglioma in L3 was resected and reconstructed with a screw and rod system; 6 months later, osteoplasty procedure by cement was done in the right posterior ilium.

Two extraadrenal pheochromocytomas were found, with no recurrence of the primary tumor (Fig. 1A). The lesions were protruding into the spinal canal and causing lumbar canal stenosis. Preoperative assessment was performed, including routine laboratory tests (electrolytes, kidney function tests, complete blood count), cardiac function (electrocardiograph (ECG), ultrasonic cardiogram (UCG), chest radiography), and endocrinological evaluation (plasma chromogranin A, metanephrines, catecholamines). Urine catecholamine was 133.71 μg/24 hour (normal: <50 μg/24 hour).

We initially planned a 2-step resection of the L3 and pelvic lesions. The patient was medically optimized with alpha- and beta-adrenergic blockers for 6 weeks, and showed reduced catecholamine and normal BP.

#### 2.1.2 The first operation

The patient was placed in the prone position under general anesthesia. We exposed the L2-L5 spinous processes and laminae, resected a 3 cm × 3 cm × 2 cm dark purple tumor mass on the right facet between L3/4 and the L3 pedicle, and performed fixation using a Moss SI screw-rod system. Perioperative BP was stable and intraoperative blood loss was 800 mL. Pathological examination was positive for chromogranin A, synaptophysin and P53, occasionally positive for S-100, but negative for melan-A and calretinin, which confirmed the diagnosis of pheochromocytoma (Fig. 1B).

The patient refused radiotherapy and was discharged after 8 days with ongoing beta-blocker medication. One month later, the urine catecholamine had reduced to 39.09 μg/24 hour and BP was normal. However, BP rose (up to 150/100 mm Hg) after cessation of antihypertensive drugs, and urine catecholamine increased to 139.50 μg/24 hour 3 months after surgery. The BP remained high (140/90 mm Hg) at the next 2-month follow-up, despite ongoing beta-blocker administration.

#### 2.1.3 The second operation

Six months later, the patient was too scared to undergo the previously designed resection of the pelvic lesion. The patient instead gave permission for percutaneous osteoplasty by polymethylmethacrylate to be performed under local anesthesia (Fig. 1C). The BP markedly increased to 200/120 mm Hg when the needle penetrated the tumor, but had decreased to normal after 2 minutes. The patient was discharged 2 days later without complication.

#### 2.1.4 Follow-up

The beta-blocker medication was gradually reduced and was stopped at 2 months postoperatively. During 2 years of follow-up, both BP and urine catecholamine were normal. There were no complications associated with osteoplasty during the follow-up period.

### 2.2 Case 2

#### 2.2.1 History and examination

**Figure 2:**
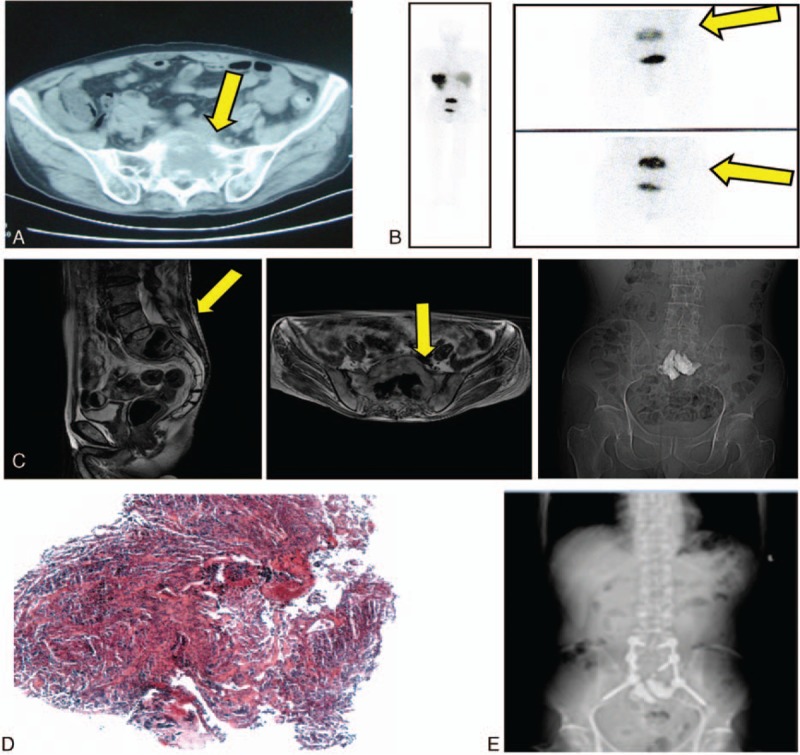
Case 2. (A) Computed tomography showing the lesion in the sacrum. (B) ^99^mTc HYNIC TOC SPECT/CT scans showing uptake in the same area. (C) The back wall of the S1 vertebral body was deficient and the cement leaked into the canal. (D) Hematoxylin and eosin-stained histological sections from the biopsy specimen confirmed extraadrenal pheochromocytoma. (E) Decompression operation was undertaken 2 months after the sacroplasty procedure.

A 58-year-old male underwent complete excision of right retroperitoneal pheochromocytoma with no metastasis 4 years previously. The patient experienced high BP (range 160–120/135–90 mm Hg) and progressive low back pain with radiating pain to the right lower limb for 1 year. Physical examination revealed a stiff lumbar region, right lower limb hyperesthesia, positive Lasegue's sign, and urine catecholamine of up to 151.24 μg/24 hour. Computed tomography and plain radiography revealed a soft mass outside the sacrum, as well as a compressed dural sac, which was confirmed by bone scanning (Fig. 2A and B). The patient was medically optimized with phenoxybenzamine and an alpha-adrenergic blocker.

#### 2.2.2 The first operation

Cement augmentation was performed to destroy the functional tumor with temporary embolization of the internal iliac artery under local anesthesia (Fig. 2C). The BP and heart rate markedly increased to 220/150 mm Hg and 140 beats/minute, respectively, when the needle penetrated the tumor, and gradually decreased to normal within 5 minutes. Pathological examination confirmed the diagnosis of pheochromocytoma (Fig. 2D). Postoperatively, the patient experienced pain relief with no improvement of leg numbness. The patient was unwilling to undergo further treatment and was discharged after 8 days.

#### 2.2.3 The second operation

The dosage of phenoxybenzamine was gradually reduced. The BP remained normal, and phenoxybenzamine was stopped 2 months later. Six months later, surgical decompression was performed to improve the impairment caused by the cement (Fig. 2E), with intraoperative blood loss of 400 mL. Nerve impairment improved to almost normal.

#### 2.2.4 Follow-up

To date, the patient has no recurrent symptoms in 6 months of follow-up. The latest urine catecholamine was 21.43 μg/24 hour. There were no complications associated with osteoplasty during the follow-up period.

## 3 Discussion

Reports of spinal pheochromocytoma are rare, 35% of which are metastases.^[2]^ Low back pain and sciatica are common presentations, with progressive hyperesthesia and sensory-motor dysfunction^[5]^; elevated BP and headache due to catecholamines are less common.^[1]^ All of these symptoms were present in the cases in the present report. Most previous cases with metastatic pheochromocytoma have undergone preoperative embolization, chemotherapy and/or radiotherapy, and/or tumor resection.^[3]^ Inspired by percutaneous ethanol and cryoablation treatment of tumor-induced osteomalacia,^[6]^ we attempted cement augmentation here for the first time. Metastatic pheochromocytoma is hypervascular and therefore carries a high risk of perioperative blood loss, generally requiring preoperative embolization.^[5]^ We minimized this risk of blood loss with a new strategy of managing intraoperative bleeding by using cement only or performing perioperative temporary embolization. Our results were similar to previous reports, in that the cement could provide adequate stability to maintain the column solidity and relieve pain.^[7]^ We further confirmed tumor destruction immediately after surgery, due to the reduction in catecholamine levels and change in related symptoms caused by cell toxicity and hyperthermic cytotoxicity of cement. Although alpha- and beta-blockers were used for preoperative adjustment, perioperative BP still increased rapidly when the needle penetrated the tumor. This increasing BP progressively decreased back to normal levels in less than 5 minutes. Continual monitoring and vasoactive preparation were needed, although blood loss was small. One postoperative complication was cement leakage into the canal and subsequent spinal cord compression. There was no sign of tumor recurrence during follow-up (2.5 and 1 year, respectively) in the present study, indicating that the local malignant tumors were eradicated.

Only 4 sacral pheochromocytomas have been reported since 1998. Coles et al^[8]^ enucleated the S3 tumor posteriorly, and the patient was disease-free after 2 years of follow-up. Another case with a tumor at S1 underwent therapy with I^131^ metaiodobenzylguanidine continually after failure of radiotherapy, and showed no tumor growth after 5 years.^[9]^ A third case with an S1–S2 lesion underwent open resection and received a total dose of 3000 cGy in 10 fractions,^[10]^ with no follow-up. In the fourth case, the sacral tumor was effectively treated with a combination of 4 methods, including preoperative sacral embolization, percutaneous cryoablation, alcohol ablation, and sacroplasty.^[11]^. In the present report, we confirmed that osteoplasty was able to relieve local pain caused by metastatic spinal pheochromocytoma in 2 cases.

To date, surgical management of spinal pheochromocytoma has remained under evaluation, with no standard criteria. We described the first 2 cases of metastatic spinal pheochromocytoma that underwent cement augmentation. In both cases, the local tumor was successfully controlled, with pain relief and decreased catecholamine levels. This might be a useful strategy to achieve rapid and sustained neurological improvements for patients with recurrence of pheochromocytoma involving the spine and the pelvis. Although there were no complications associated with osteoplasty in these 2 patients, the safety of this approach still needs to be confirmed in studies with larger sample sizes and longer follow-up periods. Surgical time, cement volume, and velocity are critical factors that need further investigation.
